# Human Rights Texts: Converting Human Rights Primary Source Documents into Data

**DOI:** 10.1371/journal.pone.0138935

**Published:** 2015-09-29

**Authors:** Christopher J. Fariss, Fridolin J. Linder, Zachary M. Jones, Charles D. Crabtree, Megan A. Biek, Ana-Sophia M. Ross, Taranamol Kaur, Michael Tsai

**Affiliations:** 1 Department of Political Science, Pennsylvania State University, University Park, PA, 16802, United States of America; 2 Department of Political Science, University of California San Diego, San Diego, CA, 92103, United States of America; University of North Carolina at Chapel Hill, UNITED STATES

## Abstract

We introduce and make publicly available a large corpus of digitized primary source human rights documents which are published annually by monitoring agencies that include Amnesty International, Human Rights Watch, the Lawyers Committee for Human Rights, and the United States Department of State. In addition to the digitized text, we also make available and describe document-term matrices, which are datasets that systematically organize the word counts from each unique document by each unique term within the corpus of human rights documents. To contextualize the importance of this corpus, we describe the development of coding procedures in the human rights community and several existing categorical indicators that have been created by human coding of the human rights documents contained in the corpus. We then discuss how the new human rights corpus and the existing human rights datasets can be used with a variety of statistical analyses and machine learning algorithms to help scholars understand how human rights practices and reporting have evolved over time. We close with a discussion of our plans for dataset maintenance, updating, and availability.

## Introduction

In this article, we introduce and make publicly available a large corpus of digitized primary source human rights documents from monitoring agencies that include Amnesty International, Human Rights Watch, the Lawyers Committee for Human Rights, and the United States Department of State. The release of these data resources is important because the human rights community has not yet taken advantage of recent advances in computational throughput, digital storage, and automated content methods. These new tools have the potential to make the analysis of large scale corpuses of primary source human rights documents not just cost effective but also informative for understanding how human rights practices and reporting have evolved over time [1]. The corpus is highly structured, which should make it useful for scholars outside the human rights community interested in the development and assessment of new statistical tools and machine learning algorithms designed for the analysis of text. In addition to a corpus that includes the raw text of human rights reports, we also introduce and make available document-term matrices (DTM), which are datasets that systematically organize the word counts from each unique document by each unique term within the corpus of human rights documents.

In the next section, we first describe the corpus of human rights texts and the DTMS we create from them. Next, we outline how human rights scholars have used these reports in the past. We then describe several existing categorical indicators that members of the human rights community have developed with human coding schemes from many of the human rights documents contained in the corpus. While each coding scheme focuses primarily on measuring state respect for “physical integrity rights”, the schemes suggest ways that scholars could use the digitized corpus to develop measures of state respect for other rights. Next, we outline possible uses for the human rights corpus and DTMs, discuss the limitations of automatic coding and human coding schemes, and provide an illustration of how automatic coding and human coding schemes can be used together to examine our corpus. Finally, we discuss our plans for dataset maintenance, updating, and availability.

## Datasets

### Corpus of Human Rights Texts

**Fig 1:**
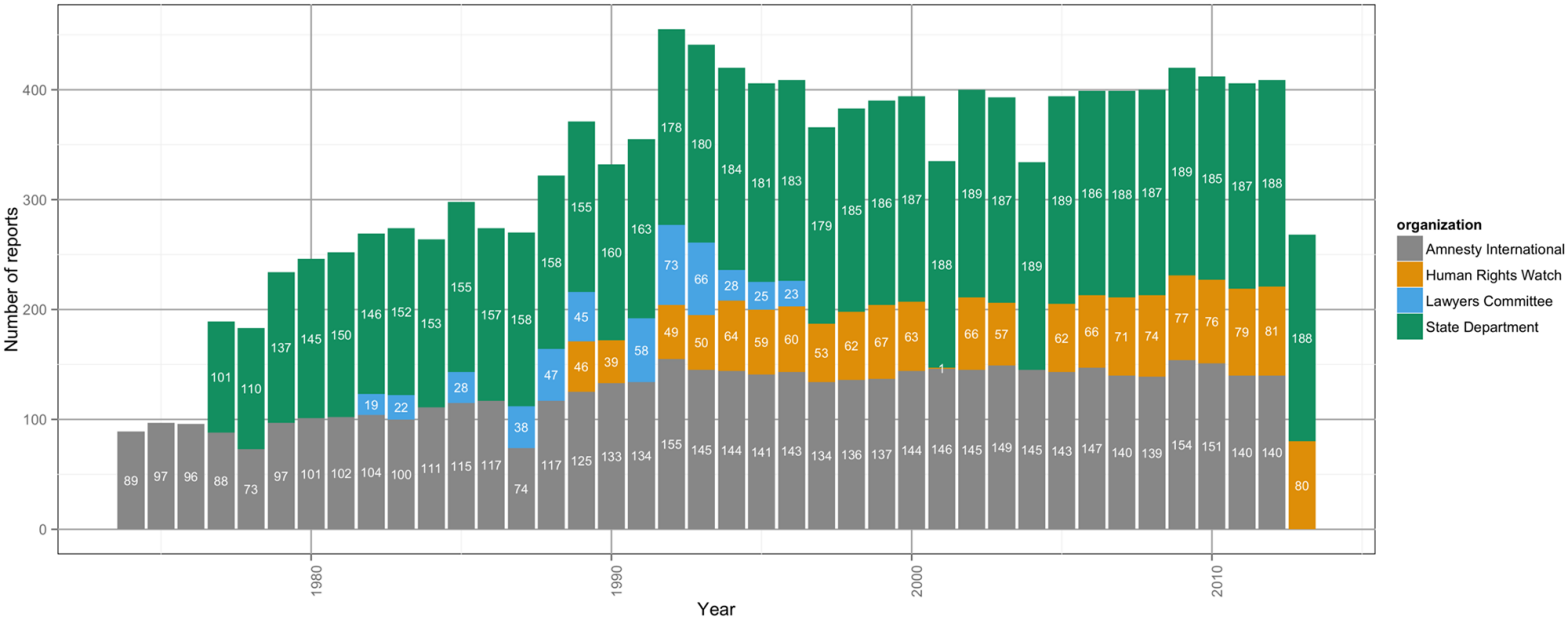
The number of human rights documents by year from the four publication sources that we have collected. The figure shows the year-by-year distribution of reports by Amnesty International (grey), Human Rights Watch (orange), the Lawyers Committee for Human Rights (blue), and the United States Department of State (green). The increasing number of reports each year coincides to both expanding coverage in the early years of the series and the increasing number of countries that enter the international state system. Some of the older documents are not easily found. We will continue to search for missing documents and eventually plan to expand this corpus to a large number of human rights publications.

**Fig 2:**
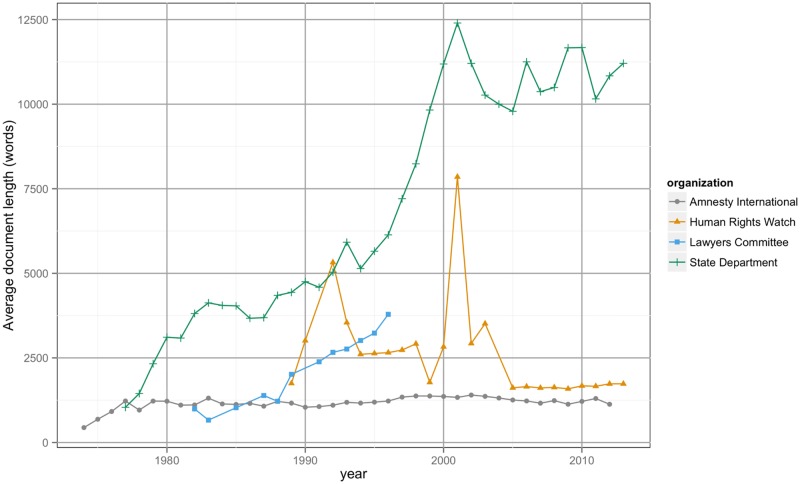
The average number of words used per human rights report by year. The figure shows the average number of words used per human rights report by year for Amnesty International (grey), Human Rights Watch (orange), the Lawyers Committee for Human Rights (blue), and the United States Department of State (green).

The corpus includes the raw text of over 14,000 human rights country reports published from four sources: Amnesty International (1974–2012), Human Rights Watch (1989–2014), the Lawyers Committee for Human Rights (1982–1996), and the United States Department of State (1977–2013). Fig 1 presents a coverage plot that indicates the temporal scope of the reports within the corpus. Fig 2 presents the average number of words per report, over time. Though all four reporting agencies share similar goals—the cataloging of human rights abuses throughout the world—each uses somewhat different methods and serves a different audience [2]. Taken together, these sources provide an increasingly detailed and accurate picture about the condition of human rights throughout the globe [1–5].

These human rights reports are the result of enormous data gathering projects that span decades. The reports contain rich qualitative information about how and to what degree states violate different types of human rights. Unfortunately, the number of individual reports coupled with the length of the reports makes it difficult for human rights scholars to analyze and discover patterns within them efficiently. Reading each published report, even for a single country, requires a tremendous investment in time. Computational methods from statistics and machine learning can help to ease the burden of reading each report by providing researchers with the means to read, analyze, and validate information from the reports automatically [6–8]. Using these methods, human rights scholars have the opportunity to discover new relationships within and between these important documentary sources.

This corpus of data also offers scholars working within the statistics and machine learning communities the opportunity to explore a new, highly structured text corpus. By highly structured, we mean that for many country-year cases, more than 1 and often 3 or 4 different documents exists and each describes the human rights conditions of the particular case. The documents are also structured in such a way that specific sections are designed to discuss specific types of human rights abuses. Additionally, human rights scholars have categorized countries across time on their respect for human rights, based on these reports. These categorizations can be used as labels for the reports and allow the use of supervised machine learning tools. We provide an example of such an application below. Overall, we hope that knowledge of this corpus from many different groups of scholars might lead to interesting new collaborations across disciplinary boundaries.

Unfortunately, until the release of our corpus, these reports have not been readily available in machine-readable format. To address this issue, we gathered the census of country year reports available from these four sources and then either (a) scanned physical reports and used optical character recognition to extract report text or (b) converted digital reports from .pdf files to raw text. We then cleaned these reports and are now making the transformed reports publicly available as a collected corpus. We hope that the open-access release of this corpus will encourage innovative human rights scholarship.

### Document-Term Matrices

In addition to publicly releasing the text files, we also created and will maintain three publicly available document-term matrices (DTM) based on the texts included in the human rights corpus. To create each DTM, let *i* = 1,…, *N* index documents and *w* = 1,…, *W* index the unique terms in the collection of documents. For each of the *i* documents, we determine the frequency of each of the unique *w* words. Each of the *D*_*iw*_ entries in a DTM represents the number of times the *w*—*th* word appears in the *i*—*th* human rights document. This procedure discards the syntax of the written content by removing any information about the order of the words. This procedure however, provides researchers opportunities to analyze the content of these human rights documents in other systematic ways, which have been shown to provide valid inferences about the content contained within text corpuses [7, 9–11].

A variety of supervised machine learning tools exist that can link the word frequencies contained within the DTMs and existing human coded categorical data [7, 8, 12]. Unsupervised statistical learning tools also exist, which are useful for revealing other patterns within the human rights document corpus [7, 13–16] without reference to the existing coded human rights variables, which we describe below. These tools are more generally part of the emergent field of computational social science or “big data” analysis [17–19] of which there are several recent examples in the study of human rights [1, 10, 20–23] and many other examples from political science and social science more generally [11, 24–28].

The first DTM we make publicly available contains almost all content from the reports. It includes the whole vocabulary of terms. However, this vocabulary does not contain the whole collection of tokens in the documents. A ‘token,’ in our case, is any combination of characters, numbers, or punctuation that is delimited by white spaces. Since the collection of all unique tokens is very large, there are several methods to decrease the size of the DTM. For the full DTM we first exclude all numbers and punctuation, since they seldom contain much meaning when separated from their syntactic context. The remaining tokens are then converted to lowercase and stemmed. Stemming reduces the number of words in the corpus by combining the counts of words that share the same basic root (e.g., “torture”, “torturing”, “tortured”, and “tortures” would all be combined into the root term “tortur”). We use the Porter stemming algorithm to accomplish this task [29]. We refer to these normalized tokens as ‘terms’ [30]. The full DTM contains information on the frequency of 170,147 unique terms in the 14,156 documents. For the second DTM, we further reduce the included vocabulary by excluding a list of very common terms or “stop words”, which are terms that often do not convey meaning (e.g., “a”, “about”, “did”, “they”, “to”, “you”) [7]. Additionally we exclude corpus specific stop words, that is words that appear in more than 95% of the documents, since these terms probably do not contain much discriminating information on the documents. This second, reduced matrix contains information on 65,707 unique terms. Finally, we release a very small matrix, which contains only the 1,000 most frequent terms from the reduced matrix.

## Existing Uses of Human Rights Reports

Since the early 1980s, social scientists have gathered and systematically coded primary source human rights documents [31–40]. The variables generated from these coding projects have been used in hundreds of published studies to understand why governments around the world choose to violate the civil and political rights of individuals (for reviews of the current and past states of this literature and extensions to it see [1, 20, 23, 41–50]. [1] provides a detailed discussion of the coding and documentation procedures of the existing datasets presented in this article as well as other sources of data used by the human rights community.

Though disagreements exist within the human rights community over conceptual definitions and coding procedures, the community—with few exceptions—is an exemplar of transparency, public access, and replicability. The human rights community, however, has not yet taken advantage of recent advances in computational throughput, digital storage, and automated content methods to leverage the rich content contained within the primary source reports used to code many existing human rights indicators. These new tools have the potential to make the analysis of large scale corpuses of primary source human rights documents not just cost effective but even more informative for understanding existing empirical puzzles.

Scholars do not need to begin analyzing the corpus or DTMs from scratch however. Many groups of political scientists have already spent considerable effort creating and validating human coded variables from the content of the human rights documents. Most but not all of these variables focus on violations of “physical integrity rights,” or “repression,” which include arrests and political imprisonment, beatings and torture, extrajudicial executions, mass killings and disappearances, all of which are practices used by political authorities against those under their jurisdiction. See [1, 43, 49, 51], or [50] for more information about this definition and its usage by human rights scholars.

Below we introduce and review several existing indicators of state respect for human rights, which are based on qualitative reading and assessment of the content within the human rights reports. We present these datasets because they are examples of how scholars have used the documents in our corpus. They also provide an entry point to scholars interested in understanding the relationship between the raw content contained in the reports and the coding schemes and resulting categorical indicators developed by a number of different political science research teams. We first review the Political Terror Scale (PTS), which was originally coded by [31, 37, 38], extended by [40] and now made available by [52]. Next we review the CIRI human rights variables, which are a set of categorical indicators introduced by [32] and [53]. Finally we consider the Hathaway Torture Scale, a categorical measures designed to assess the level of torture in each country report [39].

### Political Terror Scale Coding (1976–2013)

The PTS data are two standards-based, 5-point ordinal scales that are respectively measured from the content of the country reports published annually by the US State Department and Amnesty International respectively. See [38, 54], and [55] for additional discussion of the development of these two indices. The PTS team codes two variables which each make use of content contained within in the documents published annually by Amnesty International and the United States Department of State respectively. For each year of the series, two 5-point ordinal scales exist, with one notable exception. The PTS team did not produce the ordinal scale from the content of the Amnesty International reports in 2013 because Amnesty did not release a report for that year. The digitized text produced as part of this project may help to provide more information on any changes in the quality of the Amnesty reports over time.

The following five categorical definitions are used to score country-year units based of the content from the US State Department and Amnesty International reports:

**Level 1:** Countries under a secure rule of law, people are not imprisoned for their view, and torture is rare or exceptional. Political murders are extremely rare.
**Level 2:** There is a limited amount of imprisonment for nonviolent political activity. However, few people are affected and torture and beatings are exceptional. Political murder is rare.
**Level 3:** There is extensive political imprisonment, or a recent history of such imprisonment. Execution or other political murders and brutality may be common. Unlimited detention, with or without a trial, for political views is accepted.
**Level 4:** The practices of level 3 are expanded to larger numbers. Murders, disappearances, and torture are a common part of life. In spite of its generality, terror on this level primarily affects those who interest themselves in politics or ideas.
**Level 5:** The terrors of level 4 have been expanded to the whole population. The leaders of these societies place no limits on the means or thoroughness with which they pursue personal or ideological goals [56].

See Table 1 for the ten most important words for each category of the variable based on the reports published by Amnesty International and Table 2 for the ten most important words for each category of the variable based on the reports published by the US State Department using methods developed by [57] and described in detail below.

**Table 1:** Ten most important words for the PTS Amnesty International variable.

	1	2	3	4	5
1	polic	polic	prison	kill	kill
2	death	offic	arrest	tortur	forc
3	offic	sentenc	polit	forc	arm
4	court	illtreat	amnesti	member	group
5	concern	death	trial	arm	civilian
6	alleg	court	releas	includ	human
7	illtreat	alleg	imprison	human	secur
8	appeal	law	sentenc	execut	disappear
9	servic	servic	charg	group	attack
10	committe	concern	conscienc	arrest	execut

**Table 2:** Ten most important words for the PTS State Department variable.

	1	2	3	4	5
1	right	law	prison	kill	forc
2	law	ha	offici	forc	kill
3	provid	provid	presid	secur	secur
4	respect	gener	opposit	state	civilian
5	freedom	public	howev	dure	militari
6	employ	constitut	parti	militari	area
7	women	polic	hi	continu	continu
8	prohibit	court	author	group	group
9	constitut	employ	arrest	arrest	attack
10	public	women	offic	accord	section

### CIRI Human Rights Variables (1981–2011)

The CIRI human rights variables are a set of 3-point categorical indicators introduced by [32] and [53]. Four of these variables represent physical integrity rights and seven variables represent empowerment rights. Each CIRI human rights variable measures the level of violation on an ordinal scale where, 2 indicates that the right is not violated, 1 indicates that the right is violated occasionally, and 0 indicates that the right is violated frequently. Notice that the high values of the CIRI variables measure the highest level of respect for a specific right, whereas the lowest value on the two PTS indices capture the highest level of respect. All of the CIRI variables make use of content contained within in the documents published annually by Amnesty International and the United States Department of State. Content is taken from both two sets of reports and used together to generate each of the final country-year scores.

The CIRI coding rules attempt to use count based information from the content of the reports to rate each of the variables on one of the 3 levels (0, 1, and 2) using the following cut offs:

**Level 0:** Practiced Frequently
**Level 1:** Practiced occasionally
**Level 2:** Have not occurred / Unreported

According to the coder guidelines from the CIRI code book [33], the following terms help coders map information from the report to the appropriate score:

- Instances where violations are described by adjectives such as “gross,” “widespread,” “systematic,” “epidemic,” “extensive,” “wholesale,” “routine,” “regularly,” or likewise, are to be coded as a ZERO (have occurred frequently).
- In instances where violations are described by adjectives such as “numerous,” “many,” “various,” or likewise, you will have to use your best judgment from reading through the report to decide whether to assign that country a ONE (have occurred occasionally) or a ZERO (have occurred frequently). Look for language indicating a pattern of abuses; often, these cases merit a ZERO.

#### CIRI Physical Integrity Variables (1981–2011)

The following descriptions of the four individual physical integrity variables and the physical integrity scale are taken directly from the [33] code book and discussed at length in [32]:

**Extrajudical Killing:** The variable measuring political and other extrajudicial killings/arbitrary or unlawful depravation of life is coded as a 0 when this practice has occurred frequently in a given year; a score of 1 indicates that extrajudicial killings were practiced occasionally; and a score of 2 indicates that such killings did not occur in a given year. See Table 3 for the ten most important words for each category of this variable using methods developed by [57] and described in detail below.
**Disappearance:** The variable measuring disappearance is coded as a 0 when this practice has occurred frequently in a given year; a score of 1 indicates that disappearances occasionally occurred; and a score of 2 indicates that disappearances did not occur in a given year. See Table 4 for the ten most important words for each category of this variable using methods developed by [57] and described in detail below.
**Torture:** The variable measuring torture and other cruel, inhumane, or degrading treatment or punishment is as coded as a 0 when this practice occurred frequently in a given year; a score of 1 indicates that torture was practiced occasionally; and a score of 2 indicates that torture did not occur in a given year. See Table 5 for the ten most important words for each category of this variable using methods developed by [57] and described in detail below.
**Political Imprisonment:** The variable measuring political imprisonment is coded as a 0 when many people were imprisoned because of religious, political, or other beliefs in a given year; a score of 1 indicates that a few people were imprisoned; and a score of 2 indicates that no persons were imprisoned for any of the above reasons in a given year. See Table 6 for the ten most important words for each category of this variable using methods developed by [57] and described in detail below.

**Table 3:** Ten most important words for the CIRI Extrajudicial Killing variable.

	0	1	2
1	kill	polic	law
2	forc	prison	provid
3	secur	offici	right
4	state	case	employ
5	militari	opposit	respect
6	civilian	presid	freedom
7	area	arrest	prohibit
8	group	did	public
9	arm	ngo	constitut
10	attack	parti	women

**Table 4:** Ten most important words for the CIRI Disappearance variable.

	0	1	2
1	forc	kill	law
2	kill	arrest	provid
3	militari	secur	court
4	civilian	forc	polic
5	secur	militari	public
6	human	tortur	employ
7	group	member	prohibit
8	area	human	women
9	member	reportedli	constitut
10	arm	continu	right

**Table 5:** Ten most important words for the CIRI Torture variable.

	0	1	2
1	kill	law	law
2	forc	provid	right
3	secur	gener	freedom
4	tortur	employ	provid
5	continu	public	respect
6	arrest	women	ha
7	human	constitut	employ
8	militari	labor	public
9	reportedli	ha	women
10	dure	court	prohibit

**Table 6:** Ten most important words for the CIRI Political Imprisonment variable.

	0	1	2
1	arrest	presid	law
2	secur	opposit	polic
3	prison	howev	right
4	polit	elect	provid
5	kill	local	respect
6	detain	ngo	constitut
7	releas	offici	gener
8	forc	parti	women
9	reportedli	presidenti	offic
10	tortur	region	prohibit

#### CIRI Empowerment Rights Variables (1981–2011)

The following descriptions of the seven individual CIRI empowerment rights variables are taken directly from the [33] code book and discussed at length in [53]:

**Freedom of Assembly and Association:** This variable measuring freedom of assembly and association is coded 0 when the rights to freedom of assembly or association were severely restricted or denied completely to all citizens; a score of 1 indicates that these rights were limited for all citizens or severely restricted or denied for select groups; and a score of 2 indicates that these rights were virtually unrestricted and freely enjoyed by practically all citizens in a given year. See Table 7 for the ten most important words for each category of this variable using methods developed by [57] and described in detail below.
**Freedom of Domestic Movement:** The variable measuring freedom of domestic movement is coded as a 0 when a country severely restricts citizens’ freedom of domestic movement, or routinely restricts the movement of a significant number of citizens based on their ethnicity, gender, race, religion, marital status, political convictions, or membership in a group; a score of 1 indicates that a country places modest restrictions on the freedom of domestic movement; and a score of 2 indicates that a country does not restrict domestic movement. See Table 8 for the ten most important words for each category of this variable using methods developed by [57] and described in detail below.
**Freedom of Foreign Movement and Travel:** The variable measuring freedom of foreign movement and travel is coded as a 0 when a country restricts all or nearly all the foreign travel of its citizens; a score of 1 indicates that a country places modest restrictions on the freedom of foreign movement and travel of its citizens; and a score of 2 indicates that a country does not restrict foreign movement and travel. See Table 9 for the ten most important words for each category of this variable using methods developed by [57] and described in detail below.
**Freedom of Speech and Press:** The variable measuring freedom of speech and press is coded as a 0 when there is complete country censorship or ownership of the media; a score of 1 indicates that the country places some restrictions yet does allow limited rights to freedom of speech and the press; and a score of 2 indicates that the freedom to speak freely and to print opposing opinions without the fear of prosecution exists within a country. See Table 10 for the ten most important words for each category of this variable using methods developed by [57] and described in detail below.
**Worker Rights:** The variable measuring worker rights captures if a government systematically violates either (1) the right of association and (2) the right to organize and bargain collectively; a score of 1 indicates that a government generally protects these rights but that there are occasional violations of these rights or that there are other significant violations of worker rights; and a score of 2 indicates that governments consistently protect the exercise of these rights and that there are no mentions of violations of other worker rights. See Table 11 for the ten most important words for each category of this variable using methods developed by [57] and described in detail below.
**Electoral Self-determination:** The variable measuring electoral self-determination is coded as a 0 when the right to self-determination through political participation does not exist either in law or in practice; a score of 1 indicates that citizens have the legal right to self-determination, but that there are there are some limitations in practice that impede citizens from fully exercising this right fully; and a score of 2 indicates that citizens have the right to self-determination under the law, and exercise this right in practice through periodic, free, and fair elections held on the basis of universal suffrage. See Table 12 for the ten most important words for each category of this variable using methods developed by [57] and described in detail below.
**Freedom of Religion:** The variable measuring freedom of religion is coded as a 0 when a government engages in severe and widespread restrictions of religious freedom; a score of 1 indicates a government places moderate restrictions on religion; and a score of 2 indicates that restrictions on religious practice are practically absent within a country. See Table 13 for the ten most important words for each category of this variable using methods developed by [57] and described in detail below.

**Table 7:** Ten most important words for the CIRI Freedom of Assembly and Association variable.

	0	1	2
1	polit	polic	right
2	prison	kill	polic
3	arrest	dure	law
4	foreign	howev	provid
5	secur	court	respect
6	detain	ngo	gener
7	offici	children	constitut
8	releas	presid	investig
9	sentenc	isra	offic
10	parti	attack	labor

**Table 8:** Ten most important words for the CIRI Freedom of Domestic Movement variable.

	0	1	2
1	forc	parti	law
2	secur	opposit	right
3	reportedli	presid	polic
4	foreign	howev	provid
5	arrest	secur	investig
6	religi	arrest	case
7	offici	kill	offic
8	continu	releas	court
9	author	member	respect
10	detain	detain	gener

**Table 9:** Ten most important words for the CIRI Freedom of Foreign Movement and Travel variable.

	0	1	2
1	prison	opposit	right
2	secur	secur	polic
3	arrest	parti	law
4	polit	presid	provid
5	foreign	section	investig
6	reportedli	detain	offic
7	islam	howev	case
8	sentenc	polit	labor
9	religi	religi	respect
10	forc	releas	gener

**Table 10:** Ten most important words for the CIRI Freedom of Speech and Press variable.

	0	1	2
1	arrest	polic	right
2	polit	case	polic
3	prison	court	provid
4	secur	offic	law
5	offici	investig	respect
6	reportedli	law	labor
7	foreign	kill	constitut
8	religi	presid	gener
9	detain	roma	worker
10	sentenc	constitut	women

**Table 11:** Ten most important words for the CIRI Worker Rights variable.

	0	1	2
1	offici	polic	right
2	arrest	children	ha
3	polit	offic	respect
4	prison	law	constitut
5	foreign	case	law
6	religi	gener	freedom
7	reportedli	provid	union
8	sentenc	child	provid
9	islam	howev	thi
10	author	investig	women

**Table 12:** Ten most important words for the CIRI Electoral Self-determination variable.

	0	1	2
1	polit	opposit	polic
2	foreign	dure	right
3	arrest	presid	law
4	prison	parti	provid
5	secur	elect	offic
6	reportedli	howev	investig
7	religi	ngo	respect
8	offici	polic	labor
9	sentenc	kill	percent
10	detain	case	case

**Table 13:** Ten most important words for the CIRI Freedom of Religion variable.

	0	1	2
1	religi	state	right
2	offici	law	polic
3	reportedli	court	labor
4	islam	ethnic	provid
5	foreign	traffick	constitut
6	author	secur	respect
7	sentenc	palestinian	union
8	hi	feder	investig
9	detain	dure	law
10	arrest	parliament	gener

### Hathaway Torture Scale Coding (1985–1999)

The Hathaway Torture Scale [39] is a 5-point ordered scale for torture violations. Unlike either the PTS or CIRI variables, the [39] data relies exclusively on content from the US State Department reports. The reports are coded as follows:

**Level 1:** There are no allegations or instances of torture in this year. There are no allegations or instances of beatings in this year; or there are only isolated reports of beatings by individual police officers or guards all of whom were disciplined when caught.
**Level 2:** At least one of the following is true: There are only unsubstantiated and likely untrue allegations of torture; there are “isolated” instances of torture for which the government has provided redress; there are allegations or indications of beatings, mistreatment or harsh/rough treatment; there are some incidents of abuse of prisoners or detainees; or abuse or rough treatment occurs “sometimes” or “occasionally.” Any reported beatings put a country into at least this category regardless of government systems in place to provide redress (except in the limited circumstances noted above).
**Level 3:** At least one of the following is true: There are “some” or “occasional” allegations or incidents of torture (even “isolated” incidents unless they have been redressed or are unsubstantiated (see above)); there are “reports,” “allegations,” or “cases” of torture without reference to frequency; beatings are “common” (or “not uncommon”); there are “isolated” incidents of beatings to death or summary executions (this includes unexplained deaths suspected to be attributed to brutality) or there are beatings to death or summary executions without reference to frequency; there is severe maltreatment of prisoners; there are “numerous” reports of beatings; persons are “often” subjected to beatings; there is “regular” brutality; or psychological punishment is used.
**Level 4:** At least one of the following is true: Torture is “common”; there are “several” reports of torture; there are “many” or “numerous” allegations of torture; torture is “practiced” (without reference to frequency); there is government apathy or ineffective prevention of torture; psychological punishment is “frequently” or “often” used; there are “frequent” beatings or rough handling; mistreatment or beating is “routine”; there are “some” or “occasional” incidents of beatings to death; or there are “several” reports of beatings to death.
**Level 5:** At least one of the following is true: Torture is “prevalent” or “widespread”; there is “repeated” and “methodical” torture; there are “many” incidents of torture; torture is “routine” or standard practice; torture is “frequent”; there are “common,” “frequent,” or “many” beatings to death or summary executions; or there are “widespread” beatings to death [39].

See Table 14 for the ten most important words for each category of this variable using methods developed by [57] and described in detail below.

**Table 14:** Ten most important words for Hathaway torture variable.

	1	2	3	4	5
1	right	law	presid	forc	kill
2	law	provid	prison	secur	tortur
3	freedom	women	labor	kill	militari
4	respect	public	member	soviet	mani
5	provid	constitut	nation	polic	state
6	employ	ethnic	polit	state	secur
7	polit	employ	arrest	militari	regim
8	practic	respect	parti	dure	area
9	women	union	opposit	mani	human
10	work	court	howev	tortur	iraq

## Corpus and DTM Uses

While the existing indicators we have reviewed enjoy wide use throughout the human rights literature, they primarily focus on one class of human rights (i.e. physical integrity rights). Given the costs in training and monitoring coders for a large corpus of text based information, human rights scholars have necessarily focused their attention on the core political and civil rights contained in the International Convention for Civil and Political Rights (ICCPR) and the Convention Against Torture (CAT), especially those relating to physical integrity rights.

The human rights reports however, contain information about a much wider range of human rights violations. This information is underused as the human rights literature contains many fewer studies that focus on the broader set of “second and third generation” rights like those contained in the International Covenant on Economic, Social, and Cultural Rights (CESCR) [50]. We believe that this paper will help scholars focus on these other rights in applied research by (1) making the corpus of human rights texts and associated DTMs publicly available and (2) by introducing scholars to text based analysis procedures, which may prove useful in systematically studying the features of these documents. Provided with the raw data of the reports in machine-readable format and DTMs, scholars will be able to create new measures of state respect for other human rights, similar to the CIRI Empowerment Rights variables [33], which we reviewed above. We hope that by making available these datasets, we will encourage the creation of additional measures such as these, which would help broaden the study of human rights beyond its current focus on physical integrity rights [43].

Beyond creating new measures, human rights scholars could use the corpus and DTMs for many other purposes, such as examining inconsistencies across reports. Sometimes the information within the documents appears consistent to readers when comparing reports on the same place and time; sometimes the information does not appear consistent [2]. For example, the documents published from the early 1980s through the mid-1990s by the Lawyers Committee for Human Rights offer a focused critique of the documents published by the US State Department. To date, no scholar has directly analyzed the critical content of the Lawyers Committee for Human Rights documents relative to the content contained with the US State Department documents. If reporting agencies consistently disagree as to the state of human rights in a country, or a set of countries, then this disagreement would represent an interesting puzzle worthy of further investigation [2].

Researchers could also use the corpus in conjunction with automatic text analysis techniques to determine how the topical attention of reports varies as a function of characteristics of U.S. domestic politics and foreign policy relationships. Scholars might then extend this line of research by exploring how the topical attention, spatial focus, and language used in the country reports changes over time or across place, as the geopolitical situation changes, as technological advances increase access to information about human rights abuses, or as standards of accountability change [1, 58].

One of the main functions of human rights reports is to generate public awareness of atrocities committed by governments. The availability of these reports in a unified machine-readable format can greatly improve their accessibility to the general public. Systems can be designed to make the addition, retrieval, and full text access of these reports feasible to a non-expert audience. Natural language processing techniques can be particularly useful in summarizing and making more accessible the wealth of information contained in the reports [30]. One application of these techniques could involve using supervised machine learning classifiers to identify atrocities. The identified events could be tagged, estimates of the number of victims could be extracted, and the results could then be visualized with the goal of providing a larger picture of the state of human rights across the globe to anyone interested. We hope that the publication of a formatted and easily accessible corpus will facilitate and stimulate the development of this sort of tool by researchers interested in human rights, but also by data scientists, software engineers, or other experts that want to use their technical expertise for an important social cause.

Though we believe that researchers and the public can benefit from using automated text analysis to analyze the corpus and DTMs, this method has several important limitations. To the extent that documents are more than the sum of the words they contain, quantitative methods such as automated text analysis might miss important information by focusing primarily on the occurrence and co-occurrence of words within a document. Partially as a result of this focus, automatic text analysis can result in higher misclassification error than human coding [26]. Moreover, automatic text analysis continues to lag behind human coders in identifying emotional states within documents [59]. The degree to which these limitations might lead to biased inferences depends upon the research question being asked and the research design tools that link theory to data. These issues suggest however, that automatic text analysis does not “eliminate the need for careful thought by researchers nor remove the necessity of reading texts” [7]. Computer coding should supplement not replace human coding. This is particularly true in the context of coding human rights abuses, where scholars must regularly rely on case-specific knowledge and their ability to synthesize multiple (and sometimes competing) reports to understand how accurately a report represents human rights practices on the ground [1, 60–63].

While each approach to coding has its limitations, they can be used together to powerful effect. To illustrate this, we use a Bayesian statistical model of word frequencies [57] and the information contained in the human coded categorical variables described above to provide a preliminary examination of our corpus. With the existing scores and this method, we extract the words that are most indicative for each category of the respective human rights coding scheme. This method allows us to estimate the probability for the occurrence of each word given a coding category. First, recall that for the DTM, we let *i* = 1,…, *N* index documents and *w* = 1,…, *W* index the unique terms in the collection of human rights documents. For each of the *i* documents, we determine the frequency of each of the unique *w* words. Each of the *D*_*iw*_ entries in a DTM represents the number of times the *w*—*th* word appears in the *i*—*th* human rights document. We now consider a model that relates the frequency of words and the human coded categories reviewed above.

Following [57], we assume a multinomial distribution for the vector of word frequencies **y_k_** of size *W* (the number of unique words) in each coding category *k* = 1,2,…, *K*,

$$\begin{array}{c} y_{k}∼MN\left(n_{k},\pi_{k}\right), \end{array}$$ (1)

where *n*_*k*_ is the number of words in **y_k_** and *π*_*k*_ is the vector of probabilities for the *W* unique words. For *π*, we assume a Dirichlet prior distribution,

$$\begin{array}{c} \pi ∼Dirichlet(\alpha ), \end{array}$$ (2)

where we use the baseline frequencies of the *W* unique words in the complete collection of documents for the *α* parameter. In this Bayesian framework, the prior distribution allows us to introduce information about the baseline frequency of words in the English language, thereby normalizing noise from unexpectedly high counts of common stop words.

The closed form solution for the posterior mean of *π*_*k*_ is then,

$$\begin{array}{c} \hat{\pi_{k}}=\frac{y_{k}+\alpha }{n_{k}+\sum_{w=k}^{W}\alpha_{w}}. \end{array}$$ (3)

The odds for each element in *π*_*k*_ are **Ω_k_** = π**_k_** ○ (1 − π**_k_**), where ○ denotes the element-wise division of the two vectors. In order to obtain the most informative words for each category, we find the words that have high odds in one category, and low odds in the other categories,

$$\begin{array}{c} \delta_{k}=log(\frac{\Omega_{k}}{\Omega }), \end{array}$$ (4)

where **Ω** are the odds across all categories. The words listed in Tables 1–14 are the highest 10 elements of each δ**_k_**. Again, see [57] for more details about this and related models of text as data.

We believe that this method can provide useful insights into how well the content of the reports maps on to existing categorical human rights indicators. Tables 1–14 provide the 10 most important words for each category of the coded human rights variables described above.

Many interesting patterns emerge from this preliminary analysis and suggest avenues for future research. For example, the top 10 words for the best category on the PTS Amnesty International variable and the PTS State Department variable are quite different (see the left columns in Tables 1 and 2). This might suggest that the two reports categorize good countries in systematically different ways. It might also be an artifact of the differential coverage between the two monitoring organizations. The US State Department covers nearly every country in the international system while Amnesty International tends to ignore small countries with exceptional human rights records. Additional analysis is required to understand these differences. Consider another illustrative example: the term ‘women’ appears in the best category for nearly all of the categorical human rights variables. This might suggest that states with the best human rights practices on average receive more attention to the rights of women. This claim, however, also requires additional exploration.

These example demonstrate that even simple algorithms based on word frequencies and existing human coded variables can capture meaningful concepts from natural language. It also suggests the potential to infer qualitative categorizations for reports that have not been qualitatively coded. Further, it shows how automatic and human coding schemes can build on and reinforce each other and provide new insights for future research. Qualitative text analysis can be used to create informative labels for the textual data and quantitative methods can then be used to broaden the scope of data that can be covered. To help facilitate the use of automated content analysis in human rights research, we plan to maintain and update the corpus of documents and associated DTMs.

## Dataset Maintenance and Availability

To encourage the use of the corpus and DTMs for research purposes, we release both datasets under a Creative Commons Attribution-NonCommercial 4.0 International license. Note that our use of the original reports falls under ‘fair use’ protections defined in the Copyright Act of 1976 (17 U.S.C. § 107), as we transform the original documents into machine-readable texts and DTMs that can be use for automated analysis. To reiterate, we are making use of the machine-readable texts and DTMs for research purposes only.

We will indefinitely maintain the raw human rights text corpus and associated DTMs, updating each of them after the publication of new monitoring reports. We will maintain hosted copies of these datasets at the Harvard Database Network https://dataverse.harvard.edu/dataverse/CJFariss, specifically at http://dx.doi.org/10.7910/DVN/IAH8OY, which will be linked to at http://humanrightstexts.org/, a companion site that we have created to host the datasets and to provide information to the scholarly community about what each contains and how they can use the corpus and DTMs in their research. On http://humanrightstexts.org/, we also plan to host and maintain programming tutorials for individuals who want to use the data but have little experience analyzing large corpuses of documents or using DTMs. All datasets will be linked to through http://cfariss.com/, which is the personal homepage of the lead author.

## Conclusion

In this article, we have introduced several new datasets that scholars can use to efficiently analyze the content of human rights reports. It is our hope that researchers from all different fields will use a variety of statistical analyses and machine learning algorithms to better understand the content of these documents and how this content has evolved over time and in response to political and reporting changes. More generally, these datasets, coupled with the existing human coding schemes, automated coding algorithms, and innovative new research designs [20, 22, 64–66], will allow scholars to more thoroughly analyze reports of human rights abuse and therefore extend and contribute to the growing human rights literature.
